# Differential Flight Muscle Development in Workers, Queens and Males of the Eusocial Bees, *Apis mellifera* and *Scaptotrigona postica*

**DOI:** 10.1673/031.010.8501

**Published:** 2010-07-01

**Authors:** Fernanda Correa-Fernandez, Carminda Cruz-Landim

**Affiliations:** Departamento de Biologia, Instituto de Biociências, Universidade Estadual Paulista (UNESP), Av. 24A n° 1515, CEP 13506-900 - Rio Claro, SP, Brazil

**Keywords:** Flight muscles, histology, morphometry, male, queen, worker

## Abstract

The flight capability of the adult eusocial bees, *Apis mellifera* L. and *Scaptotrigona postica* Latreille (Hymenoptera: Apidae), is intrinsically linked to their colonial functions, such as the nuptial flight for mating in the case of queens and males, and the exploration of new habitats for nesting and food sources in the case of workers. Flight is achieved by the contraction of indirect flight muscles that produce changes in thoracic volume and, therefore, wing movement. The purpose of this work is to examine possible differences in muscle development that may be associated with the flying activity of individuals in a given life stage considering the behavioral and physiological differences among the stages and between the two species studied. Measurements of the muscle fibers obtained from light microscopy preparations of muscle were submitted to statistical analysis in order to detect the differences at a given time, or throughout the life of the individual. The results show that muscle morphology is similar in both species, but in *A. mellifera* the muscle fibers are thicker and more numerous than in *S. postica*. Differences in the fiber thickness according to life stage in all classes of individuals of both species were detected. These results are discussed in relation to the need for flying in each life stage.

## Introduction

The flight capability of the adult bees is intrinsically linked to their activities, such as the nuptial flight for mating in queens and males and tasks outside the nest, including finding a place to establish a new nest, for workers (Michener 1974). Immature males remain inside the nest and do not fly, while mature males leave the colony for mating. Similarly, after approximately 6–8 days for *Apis mellifera* L. (Hymenoptera: Apidae: Apini), virgin queens leave the nest for the nuptial flight, and workers leave the nest by 20–25 days for foraging. Thus, flight is an activity that all individuals in the nest perform only after a certain period after emergence,.

In addition to the described differences regarding the time of flight among individuals of the colony, there are behavioral differences between bees from the Apini and Meliponini tribes, especially concerning queens mating places and the range of flight that workers can achieve. *A. mellifera* queens mate in flight and, if necessary, may perform a second nuptial flight or leave the original nest with a swarm to establish a new colony. Therefore, queens need to maintain their ability to fly after mating. In *Scaptotrigona postica* Latreille (Hymenoptera: Apidae: Meliponini), the queen does not leave the colony after mating, which usually occurs in the nest. The queen only has to fly from the mother colony to her own colony, previously prepared by workers (Nogueira-Neto, 1997). In addition, the flight capability relating to distances in the search for food is higher for *A. mellifera* than for *S. postica* (Michener 1974). Eusocial bee workers perform colony maintenance tasks based on the physiological maturation of their organs, characterizing a pattern of labor division known as age polyethism (Free 1981; Hebling et al 1964; Nogueira-Neto 1997). Tasks performed by workers may be divided into two groups: tasks performed inside the colony that do not involve flight, and tasks performed outside the colony, such as foraging, which require flight on a long term scale. Intranidal tasks are performed by younger workers, while external tasks by older workers. The transition from internal to external activities in an *A. mellifera* colony with a well-distributed worker age in the population usually occurs around 20–25 days (Winston 1987). In this context evolutionary changes in these social insects must have been affected by the environment in such way that the development of flight musculature must reflect the functional demands posed by specific tasks that different adult bees perform.

Taking into account these physiological and behavioral differences, the hypothesis explored in the present work is whether changes in life phases of the bees and their activities are reflected in the differential development of flight muscles among individuals of different colony classes, both within species and between them.

## Materials and Methods

For this study, newly-emerged (leaving the brood cell), nurse (feeding larvae or provisioning brood cells), and forager (returning from foraging) workers, virgin and fertilized, laying queens, and immature and mature males of *S. postica* and *A. mellifera* were utilized. All classes of individuals were collected from colonies in the apiary at the Institute of Biosciences of UNESP, Rio Claro Campus, São Paulo State University. The functional state of workers in the colony was used instead of their chronological age because the environment within the colony influences the age at which the workers do different tasks.

Bees were anesthetized in refrigerator and the thorax was isolated from head and abdomen, and the legs were cut off. The thorax of 3 individuals of each functional condition described were fixed in 4% paraformaldehyde in 0.1 M Na phosphate buffer, pH 7.0. Only one mated queen was examined. After dehydration, the material was embedded in Leica historesin (www.leicamicrosystems.com) according the recommendations of the manufacturer. Sections, 5 µm thick, were mounted on histological slides and stained with hematoxylin and eosin. The same procedures were used for both species.

### Morphometry

The thickness of muscle fibers from dorsum-ventral bundle was measured in longitudinal sections of the muscle using the program Leica Qwin. At least fifteen muscle fibers were measured in each individual. The results were submitted to one way ANOVA in order to verify mean values differences. A Tukey test was used to establish which differences were significant. The diameter of the myofibrils within the muscle fibers was measured in cross sections of the muscles fibers. Measurements were obtained from 3 muscle fibers of one individual of each class and in each fiber, ten myofibrils were measured. These last results were not treated statistically because the homogeneity of the measures and possibility of imprecision.

## Results

### Histology

**Figure 1.**
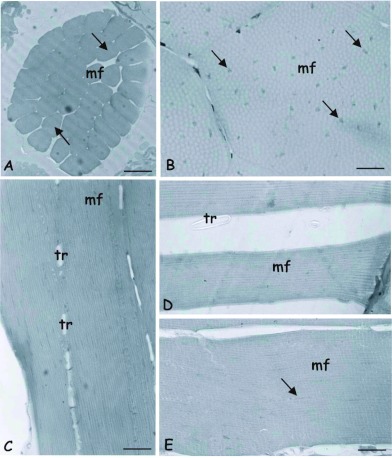
Light micrographs of bee flight muscles. A — Cross section of the muscular dorsum-ventral bundle (mf) of the indirect flight muscle of *Scaptotrigona postica*, showing the display of muscular fibers (arrows). Bar = 100µm. B - Detail of cross section of a fiber (mf) showing the myofibrils intercalated with nuclei (arrows). Bar = 20µm. C - Longitudinal section of muscle fibers of forager worker of *Apis mellifera* showing tracheae between them. Bar = 20µm. D. and E — Comparison between the widths of fibers of newly-emerged workers of *S. postica* in D and *A. mellifera* in E. tr = tracheae; arrows = mitochondria; mf= muscle fiber. Bars = 20µm. High quality figures are available online.

The general anatomy and histology of the flight muscles of *S. postica* and *A. mellifera* is similar, but the number of fibers per dorsumventral muscle bundle is larger in *A. mellifera* (± 82) than in *S. postica* (± 39). Both anterior-posterior and dorsum-ventral muscle bundles consist of large, multinuclear fibers (Figure 1 A–E). Cross sections of fibers are polygonal, approximately circular and the numerous nuclei are randomly distributed throughout the fiber, among the myofibrils, and in the fiber periphery (Figure 1 A and B). All characteristics of the morphology of muscles and muscle fibers are similar workers queens and males.

Muscles lack perimysium, as well as any connective tissue separating the fibers. Fibers are joined together by tracheal ramifications and lamellae of intracellular, amorphous substance. The tracheal branches that join the fibers appear in the sections as lacunae between them (Figure 1C). The muscular bundles are enveloped by an acellular membrane. The number of fibers forming the muscle is about twice as large in *A. mellifera* than in *S. postica*.

Comparing fibers of *S. postica* (Figure 1D) and *A. mellifera* (Figure 1E) in light micrographs made at the same magnification, a considerably difference in thickness is clearly observed.

### Morphometry

Only the thickness of the muscle fibers was measured since the fiber length did not vary during the life of the bees. Perfect cross sections of fibers were utilized to obtain measurements of myofibrils thickness (Table 1).

**Figure 2.**
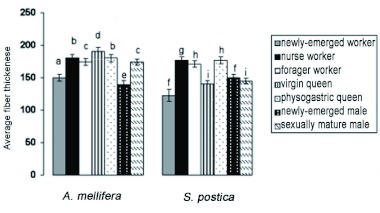
Average thickness of fibers of dorsum-ventral flight muscle bundles of workers, queens, and males of *Scaptotrigona postica* and *Apis mellifera* in different life stages. Different letters indicate statistical difference at the level of 5%. High quality figures are available online.

The average thickness of muscle fibers of *A. mellifera* and *S. postica* workers exhibit a statistically significant increase from newly-emerged to nurse worker and a significant decrease from nurse to forager worker in both species (Tables 2 and 3, Figure 2).

The average thickness of the fibers is statistically larger in *S. postica* physogastric queens but lower in *A. mellifera*. The size of the fibers in fecundated *A. mellifera* queens is statistically not different from that of nurse workers (Tables 2 and 3, Figure 2).

The average thickness of fiber muscles of newly-emerged and sexually mature males differ statistically (Tables 2 and 3, Figure 2) in both species, being larger in *A. mellifera* and smaller in *S. postica*.

Only diameter averages were calculated for the myofibrils, but Table 1 shows increases from newly-emerged to forager worker in both species, with the myofibrils slightly thicker in *A. mellifera* than in *S. postica*.

## Discussion

The results reveal that newly-emerged workers of both species have flight muscle fiber widths and myofibrils diameters smaller than those of the flying workers. Newlyemerged males of *A. mellifera* were smaller than those of the mature flying male, but this is not true of males of *S. postica* that mate within the nest rather than flying. Similarly, the virgin *A. mellifera* queen that flies to mate had larger muscle fibers than the egg-laying physogastric queen that remains in the nest. However, the virgin queen of *S. postica* that mates in the nest had smaller muscle fibers than those of the physogastric queen that flies to her new nest.

The data from workers confirm that the flight muscle at emergence is not completely developed as was before suggested by works of Herold (1965), Dickinson et al. (1998) and morphologically shown by Fernandez-Winckler and Cruz-Landim (2008).

**Table 1:**
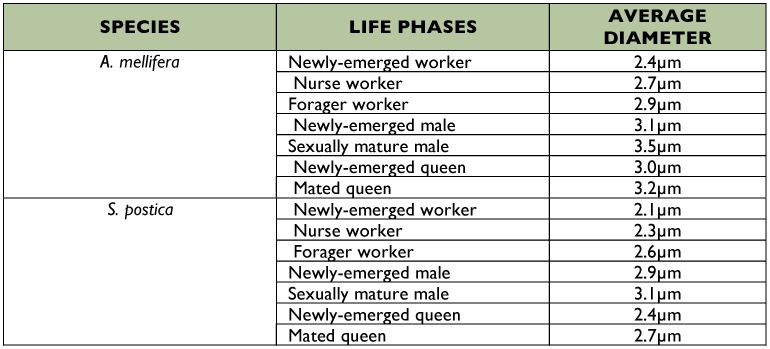
: Average diameter of myofibrils of flight muscle fibers in different life phases of bees.

**Table 2.**
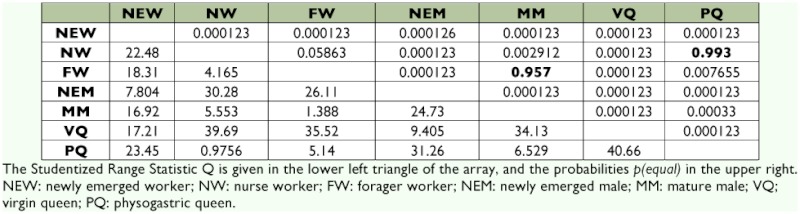
Tukey's HSD pairwise comparisons for *Abis mellifera*.

The workers perform a more diverse set of tasks than the queens and males, which are almost exclusively dedicated to reproduction. In the studied species, as in other eusocial bees, the worker functions in the colony follow a sequence based on the individual's age or physiological maturity of organs required to accomplish the task (Rosh 1930; Hebling et al. 1964; Free 1981). Part of the worker's duties are conducted inside the colony and does not require flight. Only when the worker is about 25 days old do they become foragers (Neukirch 1982; Winston 1987; Moritz 1988; Elekonich and Roberts 2005) and then need to fly. The nurse phase of the worker is immediately prior to the change for foraging activities. At this stage, the fibers of the worker flight muscles attain the largest thickness, indicating that the worker is ready for the demands of flight. The forager worker is highly developed for tasks outside of the colony, all of which are closely associated with the use of flight muscles, and therefore, an average thickness smaller than that of nurses was not expected. To this unexpected result several explanations might be found in pertinent literature. Stjernholm et al. (2005) verified a reduction in thorax mass in nectar-feeding butterflies indicating the use of thorax resources, in this case for reproduction, without affecting the flight performance negatively. Bee foragers are nectar-feeding individuals and, as in butterflies, flight muscles might be resorbed and used as supplementary source of energy. Schippers et al. (2006) showed that foragers gradually increase flight efficiency, accompanied by changes in flight metabolism. They show that some muscular proteins increase from hive bees to foragers, however the enzymes involved in aerobic performance did not increase. In accordance with this last finding, Fernandez-Winckler and CruzLandim (2008) showed that the flight muscles from foraging workers present degenerative changes in some mitochondria. Yet, as the foragers generally are the oldest workers, the possibility that the decreased size of muscle fibers might reflect a reduction in muscle mass due to age erosion or damage due to flight wear down. Nevertheless, although fiber thickness decreases in foragers, the myofibrils diameter continues to increase as already shown by Herold (1965). This might suggest that the loss of muscle material occurs within other cell organelles leaving the myofibrils that contains the contractile filaments intact and therefore don't affect flight ability.

**Table 3.**
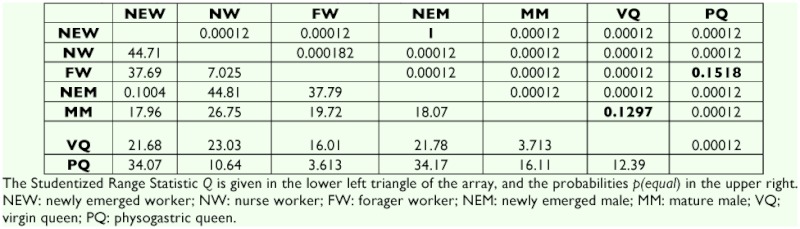
Tukey's HSD pairwise comparisons for *Scaptotrigona postica*.

The flight muscle changes are similar in both species and although the flight capability of *S. postica* workers seems to be lower, the major difference between them and *A. mellifera* seem not be in muscle fibers structure or differentiation but in the number of fibers that form the muscle bundle, that are around twice great in *A. mellifera*.

Differences also occur among the functional phases of queens and males. Queens and males remain some time after emergence inside the colony before flight to accomplish their reproductive duties. The queens of *A. mellifera* leave the colony for the nuptial flight 6 to 8 days after emergence (Winston 1987), whereas the newly-emerged virgin queens of *S. postica* do not leave the colony, neither fly outside the hive, nor perform any other task until they are required for superseding the colony's old queen or starting a new colony. The muscles of newly emerged queens of *A. mellifera* have significantly thicker fibers than the fecundated one while in *S postica* the contrary occurs. *A. mellifera* queen's muscle fibers thickness at emergence might denote a greater investment in muscle development during pupation as a preparation for the precocious flight required for fecundation. This might be achieved by the better nutrition received for this caste during larval life. Also this result reinforces the view that a certain developmental stage might be achieved before intensive flight is possible. If fiber thickness is taken as a measure of muscle development, the *A. mellifera* fecundated queen has the same developmental status as nurse worker, i. e., remain prepared for the eventual necessity of flying, as in case of swarming. The behavioral difference between the species might be refleced in flight muscle of *S. postica* whose queens mate in the nest. In this species the queens at emergence time have thinner muscle fibers than the fecundated ones. Therefore, it would be expected that the muscles of the physogastric queen of *S. postica* would degenerate, since the mated queen of this species never leaves the colony after being mated, but this did not occur, on the contrary, they were enlarged. This contrasts with the observations made in the ant *Atta sexdens* queen (Cruz-Landim and Silva-de-Moraes 1979), but might be tentatively explained by other uses of flight muscles. Meliponini queens move their wings intensely during queen-worker interactions during egg laying.

With regard to males, the differences between the studied species might also have a behavioral explanation. The males of *A. mellifera* are adapted to follow a flying queen, which would require better developed musculature for the sexually mature individuals than for *S. postica* whose mature males remain in aggregates near the colonies entrance and mate in the nest rather than in the air.

In conclusion, the present results are in accordance with the known physiological and behavioral particularities of the members of the colonies of these two species in that flight muscle development and maturation is correlated with the specific function of the individual in the colony.
